# Single-Chain Fragment Variables Targeting Leukocidin ED Can Alleviate the Inflammation of *Staphylococcus aureus*-Induced Mastitis in Mice

**DOI:** 10.3390/ijms23010334

**Published:** 2021-12-29

**Authors:** Lei Zhang, Xin Ye, Yan Jia, Manling Cheng, Dangjin Wu, Kalbinur Tohti, Jianguo Zhu

**Affiliations:** 1 Shanghai Key Laboratory of Veterinary Biotechnology, School of Agriculture and Biology, Shanghai Jiao Tong University, 800 Dongchuan Road, Shanghai 200240, China; barcelonazl@sjtu.edu.cn (L.Z.); chengmanling1113@sjtu.edu.cn (M.C.); 19981225@mail.sjtu.edu.cn (D.W.); kalbinur@sjtu.edu.cn (K.T.); 2 Laboratory of Regeneromics, School of Pharmacy, Shanghai Jiao Tong University, 800 Dongchuan Road, Shanghai 200240, China; 119170910027@sjtu.edu.cn (X.Y.); jiayan19@sjtu.edu.cn (Y.J.)

**Keywords:** mastitis, *Staphylococcus aureus* (*S. aureus*), phage surface display technology, single-chain fragment variable (scFv), bovine mammary epithelial cells (MAC-T cells), anti-inflammatory

## Abstract

*Staphylococcus aureus* is a vital bovine mastitis pathogen causing huge economic losses to the dairy industry worldwide. In our previous studies, leukotoxin ED (LukED) was detected in most *S. aureus* strains isolated from bovine mastitis. Here, four single-chain fragment variables (scFvs) (ZL8 and ZL42 targeting LukE, ZL22 and ZL23 targeting LukD) were obtained using purified LukE and LukD proteins as the antigens after five rounds of bio-panning. The complementarity-determining region 3 (CDR3) of the VH domain of these scFvs exhibited significant diversities. *In vitro*, the scFvs significantly decreased LukED-induced cell killing by inhibiting the binding of LukED to chemokine receptors (CCR5 and CXCR2) and reduced the death rates of bovine neutrophils and MAC-T cells caused by LukED and *S. aureus* (*p* < 0.05). In an *S. aureus*-induced mouse mastitis model, histopathology and MPO results revealed that scFvs ameliorated the histopathological damages and reduced the infiltration of inflammatory cells (*p* < 0.05). The ELISA and qPCR assays showed that scFvs reduced the transcription and expression levels of Tumor Necrosis Factor-alpha (TNF-α), interleukin-1β (IL-1β), IL-6, IL-8 and IL-18 (*p* < 0.05). The overall results demonstrated the protective anti-inflammatory effect of scFvs in vitro and in vivo, enlightening the potential role of scFvs in the prevention and treatment of *S. aureus*-induced mastitis.

## 1. Introduction

Mastitis is a major disease that seriously harms the dairy industry and can cause great economic losses to farmers [1,2,3,4]. *Staphylococcus aureus* (*S. aureus*) is a major opportunistic pathogen of humans and animals and one of the most pathogenic microorganisms in bovine mastitis [5,6,7]. *S. aureus* can secrete large amounts of virulence factors, such as fibronectin-binding protein A (FnBPA), coagulase (Coa), β-hemolysin (Hlb), glyceraldehyde-3-phosphate dehydrogenase (GapC) and leukotoxin [8,9,10]. The interaction between virulence factors is the main reason for the pathogenicity of *S. aureus* [1,11]. Studies have shown that *S.aureus* can cleave bovine mammary epithelial cells (MAC-T cells) and immune cells by secreting leukotoxins, resulting in the damage and inflammation of mammary gland tissues. The rapid recruitment of neutrophils to the infected site is the key to limiting *S. aureus* infection in bovine mastitis [10]. Previous studies have shown that LukMF is the most abundant leukocidin secreted by bovine mastitis isolates and plays an important role in the pathogenesis of bovine mastitis [12,13]. However, previous studies in our lab showed that the detection rate of LukED was more than 90% in *S. aureus* isolated from the dairy farm in Pudong, Shanghai and Dali, Yunnan, and significantly higher than that of LukMF (data unpublished). Some studies have revealed that LukED can effectively target and kill bovine neutrophils in vitro and in vivo and can also promote the replication of *S. aureus* in vivo and the progression of the disease by directly killing murine phagocytes recruited to the site of tissue infection [5,14,15,16]. LukED is a vital pathogenic factor, as well as a usually neglected leucotoxin, which plays an important role in the lethality of mice. Both LukMF and LukED are bicomponent pore-forming toxins, which are secreted in the form of S and F-monomers [5,17]. The S subunit binds to specific protein receptors on the cell surface and then recruits F subunits to form octamer pores by alternating oligomerization on the cell membrane, eventually resulting in cell death [16]. Previous studies have shown that LukED cleaves various cells by recognizing different receptors (CCR5, CXCR1, CXCR2 and DARC) [16,18]. These results suggested that LukED secreted by *S. aureus*, including methicillin-resistant *S. aureus* (MRSA), may play an important role in the pathogenesis and inflammatory response of mastitis. At present, almost no related studies have shown that LukED has an effect on the pathogenesis of bovine mastitis. Thus, the research of LukED is helpful to further clarify the pathogenesis of bovine mastitis.

At present, antibiotics are still the main method for the treatment of bovine mastitis. However, drug residues and the emergence of antibiotic-resistant bacterial strains restrict the use of antibiotics in economic animals [19,20,21,22]. Most of the vaccines were not effective at protecting from new infections in the treatment of bovine mastitis caused by *S. aureus* [23]. Some have been proven to exhibit a good effect on the treatment of bovine mastitis. However, the complexity and side effects of the components of herbal medicines are still unclear [24]. Studies have shown that genetically engineered antibodies provide an important means for the prevention and treatment of dairy cow mastitis. As one of the most common genetic engineering antibodies, scFv has been demonstrated to provide protection for bovine mastitis caused by *S. aureus* [8,25]. ScFv consists of a variable region of light chain (VL) and heavy chain (VH), which retains the complete antigen-binding sites of the full-length antibody [24]. Meanwhile, the characteristics of low immunogenicity, strong specificity, small size and genetic engineering are suitable for producing bacterial expression systems in large quantities [26]. ScFv is a powerful tool for the prevention and treatment of microbial diseases and has been shown to be an effective treatment for controlling bacterial and viral infections (e.g., scFv can decrease the damage of *Xanthomonas citri* and *Salmonella enteritis*, scFv provides the protection against infection of piglets caused by PEDV virus and prevents the invasion of infectious bursal disease virus (IBDV) and human influenza virus H5N1) [27,28,29]. Phage display technology was used to obtain scFv-aiming LukED in our lab. Compared with hybridoma technology, it is a cheaper and faster method to obtain scFv [30]. Now phage display technology has been increasingly used in livestock research (e.g., phage display scFv libraries generated from camel, rabbit, pig, sheep and chicken) [31,32,33,34,35]. Studies have shown that scFv targeting *S. aureus* can effectively inhibit the growth of *S. aureus* in vitro and has a good protective effect on mastitis in mice [8].

The secretion level of the pro-inflammatory cytokines is one of the prominent indicators of inflammatory response and plays a key role in the host’s defense against invasive pathogenic microorganisms [36]. Previous studies in our lab have also proven that the detection of the transcription and expression of tumor necrosis factor-alpha (TNF-α), interleukin-1β (IL-1β), IL-6, IL-8 and IL-18 can reflect the pathogenicity of *S. aureus* and the anti-inflammatory role of scFv in anti-bacterial immune response [8,25]. Appropriate levels of pro-inflammatory cytokines are important for the immune response against pathogens, but excessive production of cytokines may lead to severe tissue damage [37]. Therefore, those five pro-inflammatory cytokines were selected as the detection index in this paper.

In summary, the purpose of this study was to clarify whether scFv can affect the recognition of *S. aureus* LukED to the corresponding cell receptor, eventually resulting in cell death and the protective effect of scFv on *S. aureus*-infected MAC-T cells and mouse mammary gland tissue. These studies provide the basis for the development of genetically engineered therapeutic drugs, and these bovine scFv may be suitable candidates for the prevention and treatment of bovine mastitis caused by *S. aureus*.

## 2. Results

### 2.1. Expression and Purification of LukE and LukD

**Figure 1:**
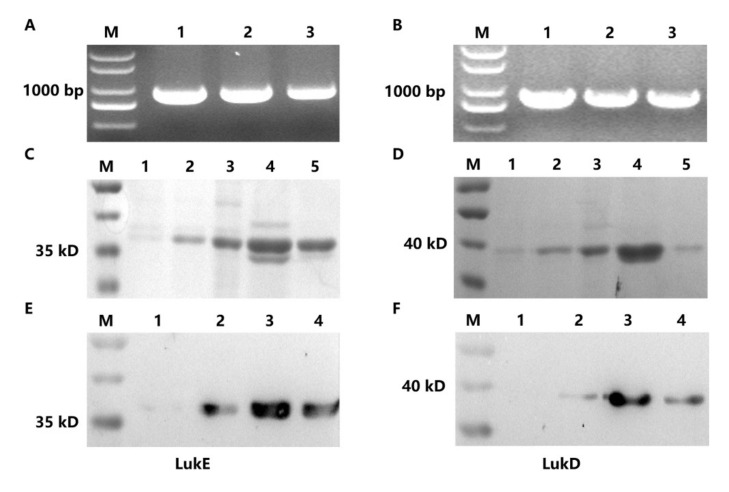
The gene fragments and protein products of leukotoxin ED of *S. aureus*. (**A**,**B**) PCR amplification products. Lane M of (**A**,**B**), 2000-bp DNA ladder; lanes 1–3 of (**A**,**B**), PCR amplification product of *lukE* (**A**), *lukD* **(B**). (**C**,**D**) The SDS–PAGE analysis. Lane M of (**C**,**D**), protein molecular weight marker; lanes 1–5 of (**C**,**D**), the purified protein of LukE and LukD eluted by 20, 40, 60, 100 and 150 mM imidazole, respectively. (**E**,**F**) The western blotting assay. Lane M of (**E**,**F**), protein molecular weight marker; lane 1 of (**E**,**F**), without IPTG induction; lanes 2–4 of (**E**,**F**), the purified protein of LukE and LukD eluted by 60, 100 and 150 mM imidazole, respectively.

The genes (Figure 1A,B) of *lukE* and *lukD* were subcloned into pET-28a (+) plasmid and then expressed on BL21 (DE3) competent *E. coli* cells, respectively. The length of *lukE* and *lukD* were approximately 900 bp. SDS-PAGE analysis revealed that the protein samples (LukE and LukD) had a molecular weight of 36 kDa and were expressed in a soluble form (Figure 1C,D). The western blotting assay exhibited a distinct single band after dyeing, and the concentration of the proteins (LukE and LukD) detected by the BCA Protein Assay Kit was approximately 450 μg/mL, suggesting that the concentration and purity of the two antigen proteins were high enough to apply in the following experiment (Figure 1E,F).

### 2.2. Screening of ScFvs Targeting LukE and LukD

**Figure 2:**
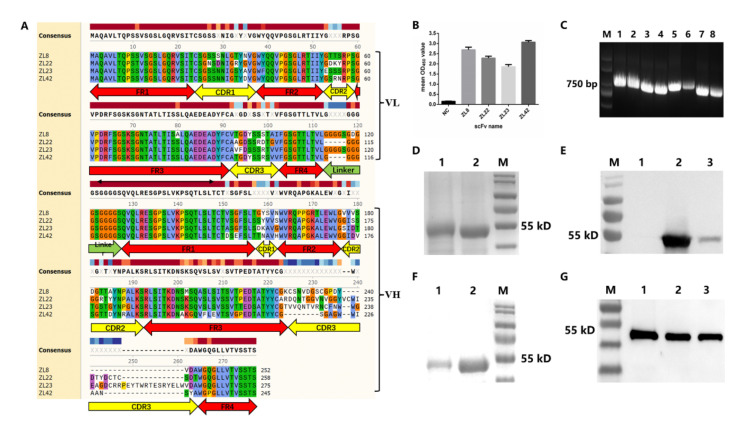
The characteristics, expression and purification of ScFvs. (**A**) Alignment of multiple protein sequences of ZL8, ZL22, ZL23 and ZL42. Four FRs and three CDRs for both VH and VL of each scFv are illustrated above. The same color regions represent the identity of amino acids, while the blank regions represent amino acid differences of the four scFvs. (**B**) The value of OD_450_ of the four clones. Lane M, 2000-bp DNA ladder. (**C**) PCR products of scFvs amplificated from positive scFv phage clones. Lane M, 2000-bp DNA ladder; lanes 1–2, scFv PCR product of ZL23; lanes 3–4, scFv PCR product of ZL8; lanes 5–6, scFv PCR product of ZL22; lanes 7–8, scFv PCR product of ZL42. (**D**,**F**) The SDS–PAGE analysis. Lane M of (**D**,**F**), protein molecular weight marker; lanes 1–2 of (**D**), the purified proteins of ZL23 and ZL22, respectively. Lanes 1–2 of (**F**), the purified proteins of ZL8 and ZL42, respectively. (**E**,**G**) The western blotting assay. Lane M of (**E**,**G**), protein molecular weight marker; lane 1 of (**E**), without IPTG induction; lanes 2–3 of (**E**), the purified protein of ZL23; lanes 1–3 of (**G**), the purified protein of ZL8, ZL22 and ZL42, respectively.

After five rounds of bio-panning, the final output titer of the scFv phage display library targeting LukE and LukD reached 2.14 × 10^8^ cfu/mL (Table 1) and 1.25 × 10^8^ cfu/mL (Table 2), which was 130-fold and 295-fold higher than that of the first round, respectively. The two scFv phage display libraries were large enough for the screening of scFvs targeting LukE and LukD. The positive clone was defined as 2.5 times (OD_450_) higher than that of the negative control. After phage ELISA, four positive clones (targeting the antigens of LukE and LukD, respectively) with higher values of OD_450_ were obtained from the scFv phage display library. The values of OD_450_ of the four clones are shown in Figure 2B.

### 2.3. Characteristics, Expression and Purification of ScFvs

Four positive clones were used as templates to amplify the four scFvs, designated as ZL8 (accession no. OL839275), ZL22 (accession no. OL839276), ZL23 (accession no. OL839277) and ZL42 (accession no. OL839278), respectively. The amino acid sequences of the four scFvs were analyzed after sequencing. The results showed that the VL and VH amino acid sequences of the four scFvs were successively joined with a short peptide linker (Figure 2A). The amino acid sequences of the VL and VH domains consist of four frame regions (FRs) and three complementary determination regions (CDRs). The FRs of scFv were highly conserved according to the alignment of multiple protein sequences. The CDRs of scFv, especially CDR3 of VH, exhibited significant diversities.

The lengths of the four scFv gene fragments were around 730–830 bp (Figure 2C). The SDS-PAGE analysis showed that the sizes of the four scFvs were approximately 54 kDa (contained 26 kDa of GST tag) and expressed in a soluble form (Figure 2D,E). The western blotting assay revealed that four scFv proteins exhibited a single band, revealing that the purity of the four scFv proteins is satisfactory for the following experiments (Figure 2F,G).

### 2.4. ScFvs Can Inhibit the Binding of LukED to CCR5 and CXCR2 In Vitro

**Figure 3:**
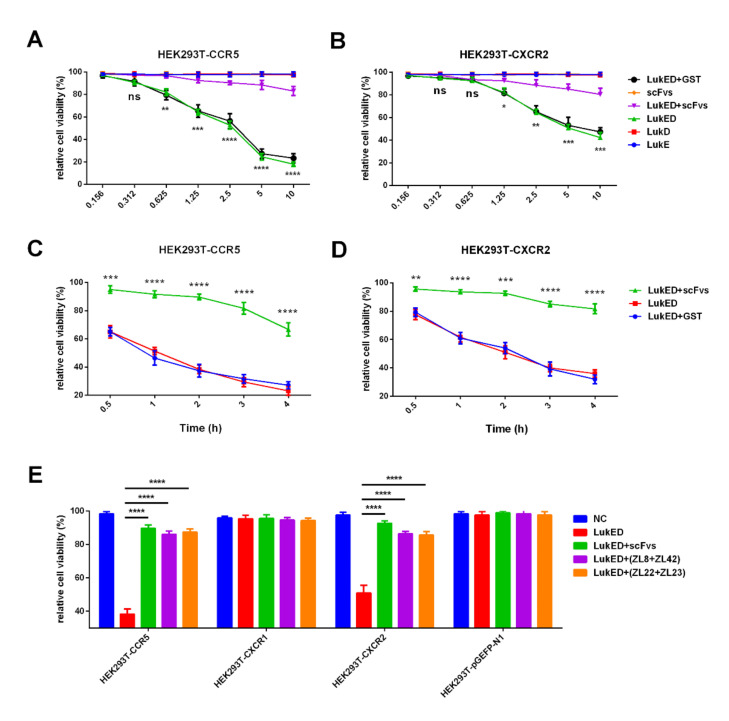
The protective effect of scFvs on the cell killing of HEK293T cells containing CCR5 and CXCR2 caused by LukED. (**A**,**B**) The cell killing of HEK293T cells containing CCR5 and CXCR2 upon incubation with the scFvs mixture and different concentrations of LukED. (**C**,**D**) The cell killing of HEK293T cells containing CCR5 and CXCR2 upon incubation with LukED and the scFvs mixture over time. (**E**) The effect of scFvs on the rise of the relative cell viability of LukED-mediated HEK293T cells by blocking the binding of LukED with CCR5 and CXCR2. * *p*, ** *p*, *** *p*, **** *p* vs. the NC group (LukED group). Data represent the mean results ± SD (*n* = 3) (ns = *p* > 0.05, * *p* < 0.05, ** *p* < 0.01, *** *p* < 0.001, **** *p* < 0.0001).

The gene products of CCR5, CXCR1 and CXCR2 were subcloned into pEGFP-N1 (data not shown) for the construction of recombinant plasmids. CCR5, CXCR1 and CXCR2 were stably transfected into HEK293T cells. The results revealed that the purified LukED, rather than LukE or LukD, lysed HEK293T cells containing CCR5 and CXCR2 in a dose and time-dependent manner (Figure 3A–D). Compared with the LukED group (10 μg/mL) toward HEK293T cells containing CCR5 or CXCR2, just about 20% of HEK293T cells were damaged in the scFvs mixture group, exhibiting three-fold and four-fold decreases, respectively (*p* < 0.05). Furthermore, HEK293T cells containing CCR5 and CXCR2, rather than CXCR1, can be lysed by LukED (Figure 3E), revealing LukED can cause cell death by direct interaction with CCR5 and CXCR2. Moreover, the scFvs groups (ZL8 + ZL42 group, ZL22 + ZL23 group and scFvs mixture group) can provide a positive effect on the rise of the relative cell viability of HEK293T cells containing CCR5 and CXCR2 compared with the cell-killing caused by LukED (*p* < 0.05).

### 2.5. ScFvs Can Effectively Weaken Cell Killing Caused by LukED

**Figure 4:**
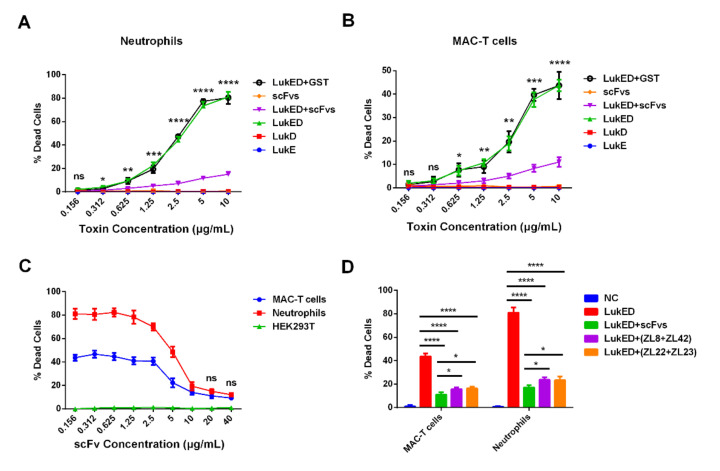
The function of scFvs is to inhibit LukED-mediated cell killing. (**A**,**B**) The cell killing of bovine neutrophils and MAC-T cells upon incubation with the scFvs mixture and different concentrations of LukED, respectively. (**C**) The protective effect of different concentrations of scFvs on the inhibition of LukED-mediated cell killing. (**D**) The protective effect of different groups of scFvs (ZL8 + ZL42 group, ZL22+ZL23 group and the scFvs mixture group) on the inhibition of LukED-mediated cell killing. * *p*, ** *p*, *** *p*, **** *p* vs. LukED group. Data represent the mean results ± SD (n = 3) (ns = *p* > 0.05, * *p* < 0.05, ** *p* < 0.01, *** *p* < 0.001, **** *p* < 0.0001). Data represent the mean results ± SD (n = 3).

Here, we evaluated the ability of LukED toward bovine immune cells (bovine neutrophils) and MAC-T cells and the protective effect of scFvs. The results revealed that the purified LukED, rather than LukE or LukD subunits, was cytotoxic to bovine neutrophils and MAC-T cells, exhibiting an increasingly higher death rate of cells along with the rise of LukED concentration. Compared with the LukED group (greater than 0.312 μg/mL), the scFvs mixture group can significantly decrease the death rate of bovine neutrophils and MAC-T cells (*p* < 0.05) (Figure 4A,B). Meanwhile, we noticed that scFvs functioned to protect bovine neutrophils and MAC-T cells from being killed in a dose-dependent manner (Figure 4C). The results also demonstrated that the scFvs of ZL8 + ZL42, ZL22 + ZL23 and the scFvs mixture groups can obviously inhibit the cell killing of bovine neutrophils (from 45% to 16% in the ZL8 + ZL42 and ZL22 + ZL23 groups, and from 45% to 11% in the scFvs mixture group) and MAC-T cells (from 80% to 24% in the ZL8 + ZL42 and ZL22 + ZL23 groups, and from 80% to 18% in the scFvs mixture group) compared with the LukED group (*p* < 0.0001), which exhibited a 3–4-fold and 3.3–4.5-fold decrease, respectively (Figure 4D). Moreover, the effect of the scFvs mixture group on the decrease of cell death rate was better than the ZL8 + ZL42 and ZL22 + ZL23 groups (*p* < 0.05).

### 2.6. ScFv Can Weaken the Cytotoxicity of MAC-T Cells and Bovine Neutrophils Caused by S. aureus

**Figure 5:**
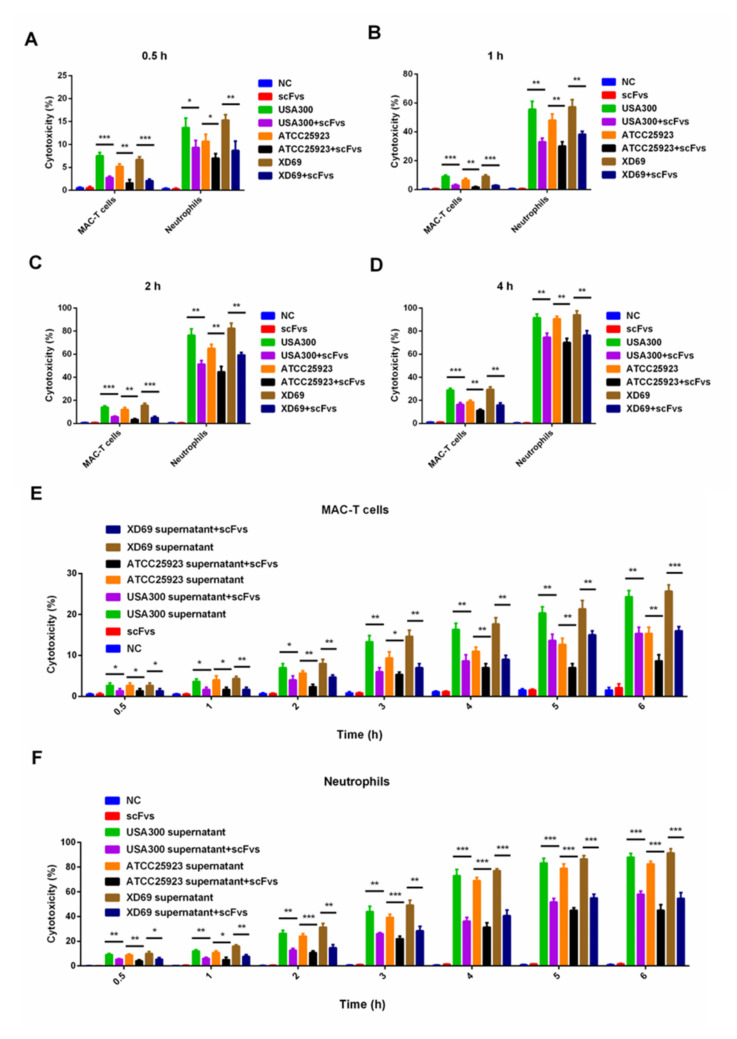
The cell killing of bovine neutrophils and MAC-T cells. (**A**–**D**) The LDH release of bovine neutrophils and MAC-T cells infected by three strains (USA300, ATCC25923 and XD69) over time. (**E**,**F**) The LDH release of bovine neutrophils and MAC-T cells infected by the supernatant of three strains over time. Data represent the mean results ± SD (*n* = 3) (ns = *p* > 0.05, * *p* < 0.05, ** *p* < 0.01, *** *p* < 0.001).

Three *S. aureus* strains (i.e., USA300, ATCC25923 and XD69) and two types of cells (bovine neutrophils and MAC-T cells) were selected to perform the assay. The results revealed that the pathogenicity of USA300 and XD69 was stronger than that of ATCC25923 (Figure 5A–F). Moreover, compared with the intoxication of USA300, ATCC25923 or XD69 groups, the cytotoxicity of bovine neutrophils and MAC-T cells treated with the scFvs group was significantly reduced over time (*p* < 0.05) (Figure 5A–D). Furthermore, after a 6-h incubation of the supernatant of *S. aureus* with scFvs, the death rate of MAC-T cells intoxicated by the supernatant decreased from 25% to 15% in the USA300 group (*p* < 0.01), from 16% to 8% in the ATCC25923 group (*p* < 0.01) and from 26% to 16% in the XD69 group (*p* < 0.001), exhibiting 1.6–2-fold decreases (*p* < 0.05) (Figure 5E). Similarly, the LDH release of bovine neutrophils intoxicated by the supernatant of USA300 (from 90% to 60%) (*p* < 0.001), ATCC25923 (from 80% to 45%) (*p* < 0.001) and XD69 (from 90% to55%) (*p* < 0.001) decreased significantly after incubation with the mixture of four scFvs after a 6-h incubation with scFvs (Figure 5F).

### 2.7. ScFv Can Inhibit the S. aureus Proliferation In Vivo

**Figure 6:**
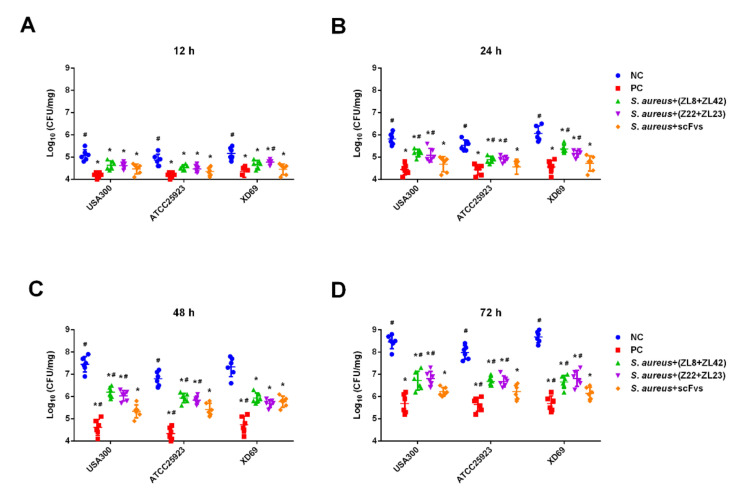
Bacteria proliferation assay. (**A**–**D**) The proliferation of the three strains were inhibited by the scFvs mixture in mammary gland tissues over time. Data represent the mean results ± SD (*n* = 6). # *p* < 0.05 vs. the scFvs mixture group. * *p* < 0.05 vs. the NC group.

To evaluate the specific contribution of *S. aureus* to local infection over time, we monitored the bacterial proliferation in mammary gland tissues infected with three *S. aureus* strains (USA300, ATCC25923 and XD69) (Figure 6A–D). The results showed that after infection for 12 h, the bacterial burden in the USA300, ATCC25923 and XD69 groups was significantly increased compared with the scFvs mixture group (*p* < 0.05) (Figure 6A). After infection for 72 h, the bacterial burden of mammary gland tissues infected by the three *S. aureus* strains reached more than 1 × 10^8^ CFU/mL, which exhibited a 1000-fold increase compared with the infection for 12 h (Figure 6D). Comparatively, the bacterial burden in the scFvs mixture group only exhibited a 100-fold increase from 12 to 72 h. The results also showed that the scFvs mixture group had a better effect on the inhibition of bacteria proliferation compared with the ZL8 + ZL42 and ZL22 + ZL23 groups at 24, 48 and 72 h (*p* < 0.05) (Figure 6B–D).

### 2.8. Histopathologic Changes and MPO Activity in Mammary Gland Tissues

**Figure 7:**
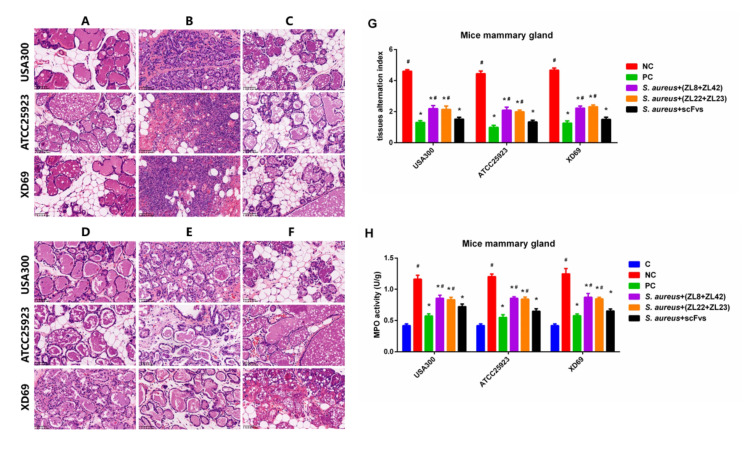
Functions of scFvs on histopathological changes and bovine neutrophils infiltration in *S. aureus*-induced mammary gland tissues. (**A**) Control group. (**B**) NC group. (**C**) PC group. (**D**) ZL8+ZL42 group. (**E**) ZL22 + ZL23 group. (**F**) scFvs mixture group. (**G**) Tissue alteration index of mammary gland tissues. (**H**) MPO activity assay of mammary gland tissues. Data represent the mean results ± SD (*n* = 6). # *p* < 0.05 vs. the scFvs mixture group. * *p* < 0.05 vs. the NC group.

H & E staining and microscopic observation were performed to evaluate the pathological section of mammary gland tissues. The results showed that no histopathological changes were observed in the control group (Figure 7A). The mammary gland tissue structures infected with the NC group showed severe histopathologic changes, such as damaged mammary epithelial cells and mammary acini, broken lobuli mammae and infiltrated inflammatory cells (Figure 7B). However, these histological changes in the scFvs mixture groups were significantly ameliorated (*p* < 0.05) compared to the NC group (Figure 7C). The data also showed that there is no difference between the scFvs mixture group and the PC group, which exhibited very slight inflammatory injury and mild inflammatory cells interstitial infiltration (Figure 7C,F). The mammary gland tissue alternation index showed that the scFvs mixture group was better than the ZL8+ZL42 and ZL22+ZL23 groups on the protection of the mammary gland tissues (Figure 7G). Moreover, the MPO activity exhibited similar results (Figure 7H). Moreover, there was no difference between the scFvs mixture group and the PC group in the treatment of mammary gland tissues.

### 2.9. ScFvs Can Decrease the Production of Inflammatory Cytokine

**Figure 8:**
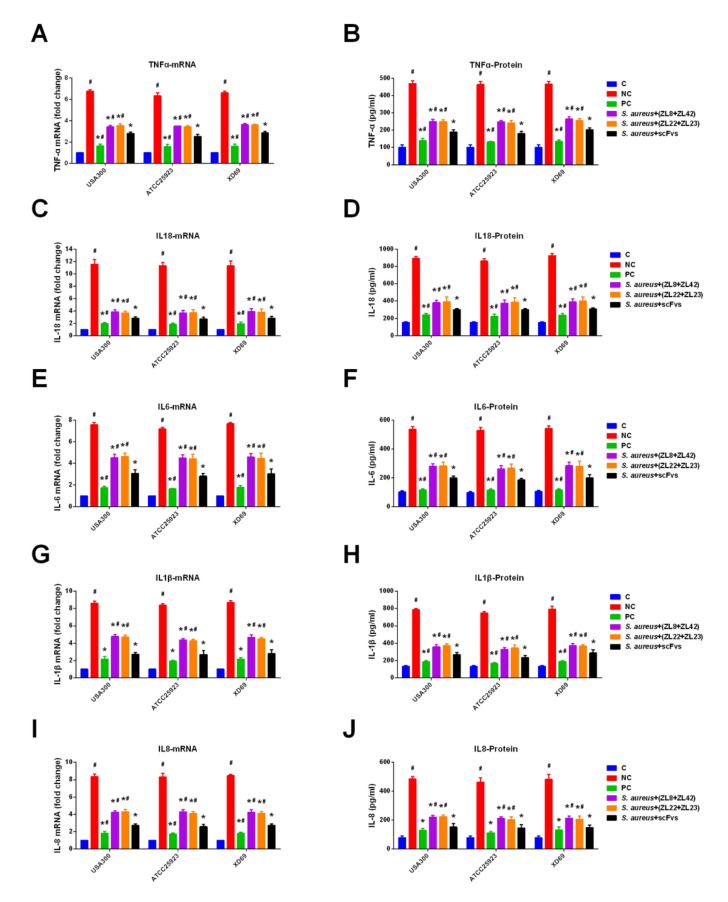
The effect of scFvs on the production of five pro-inflammatory cytokines in mammary gland tissues. (**A**,**C**,**E**,**G**,**I**) The transcription levels of TNF-α, IL-18, IL-6, IL-1β and IL-8 in mammary gland tissues infected by three strains, respectively. (**B**,**D**,**F**,**H**,**J**) The expression level of TNF-α, IL-18, IL-6, IL-1β and IL-8 in mammary gland tissues infected by three strains, respectively. *GAPDH* was used as the control gene. Data represent the mean results ± SD (*n* = 6). # *p* < 0.05 vs. the scFvs mixture group. * *p* < 0.05 vs. the NC group.

The production of pro-inflammatory cytokines can reflect the severity of inflammation caused by *S. aureus.* The data showed that compared with the NC group, the transcription levels of TNF-α, IL-18, IL-6, IL-1β and IL-8 in mammary gland tissues were significantly inhibited in the scFvs mixture group (*p* < 0.05) (Figure 8A,C,E,G,I), especially IL-18, exhibiting a 4-fold decrease (reduced from 12 to 3). Moreover, the expression levels of five pro-inflammatory cytokines in the scFvs mixture group were lower than that in the NC group (*p* < 0.05) (the concentration decreased approximately from 490 to 190 pg/mL in the TNF-α group, from 890 to 300 pg/mL in the IL-18 group, from 540 to 200 pg/mL in the IL-6 group, from 780 to 270 pg/mL in the IL-1β group and from 480 to 150 pg/mL in the IL-8 group) (Figure 8B,D,F,H,J). Moreover, the results also indicated that the ZL8 + ZL42 and ZL22 + ZL23 groups could dramatically reduce the transcription and expression levels of five inflammatory cytokines compared to the NC group (*p* < 0.05). It was noticed that the scFvs mixture group showed a better protective effect than the ZL8 + ZL42 and ZL22 + ZL23 groups on the production of five pro-inflammatory cytokines (*p* < 0.05).

## 3. Discussion

Mastitis is defined as an inflammatory response of mammary gland tissue infection. *S. aureus* is one of the most vital zoonotic bacterial pathogens causing mastitis in dairy cows worldwide [38]. Studies have shown that leukotoxin secreted by *S. aureus* plays a major role in the pathogenesis of mastitis. The killing effect of *S. aureus* on cells and mice varied significantly according to the source of the strains [12]. Previous studies have shown that *S. aureus* with specific genotypes (i.e., USA300) exhibited an increase in cross-infection between cattle and humans [39]. Therefore, we selected three *S. aureus* strains and their culture supernatants to verify the pathogenicity of *S. aureus* isolated from different sources. At present, there is no efficient treatment for bovine mastitis. Previous studies in our lab have shown that scFvs targeting different antigens had a significant protective effect on mastitis in mice [8,25,40]. In brief, we obtained four scFvs (ZL8 and ZL42 aimed at LukE, ZL22 and ZL23 aimed at LukD) using the phage display technique. The results showed that the pathogenicity of the USA300 and XD69 strains was stronger than that of the ATCC25923 strain. Moreover, the results confirmed that these four scFvs had protective effects on MAC-T cells, bovine neutrophils and mammary gland tissues infected by *S. aureus*.

The positive rate of the phage display library was increased by reducing the concentration of coated antigen and increasing the washing times in subsequent rounds of bio-panning. The results showed that after five rounds of bio-panning, the final output titers of the scFv phage display libraries for LukE and LukD reached 2.14 × 108 (Table 4) and 1.25 × 108 cfu/mL (Table 5), respectively, which was 130 and 295-fold higher than that in the first round (1.65 × 10^6^ and 4.25 × 10^5^ cfu/mL, respectively). Previously, Wang et al. reported that the final output titer of the scFv phage display library was 2.32 × 10^7^ cfu/mL, which was 9.2-fold and 5.4-fold lower than that (2.14 × 10^8^ cfu/mL in the LukE group and 1.25 × 10^8^ cfu/mL in the LukD group) in this study [25]. Zhang et al. reported that the final output titer of the scFv phage display library was 1.4 × 10^8^ cfu/mL, which was similar to that in this study [8]. Four scFvs were obtained using phage ELISA. The results of the amino acid sequence alignment of scFvs showed that the CDRs, which were probably related to the antigen-binding site, exhibited high variability in amino acid composition and quantity, suggesting that the scFvs obtained in this study may have different epitope specificity.

In order to prove whether scFv can affect the binding of LukED to the receptor on the cell membrane, thus affecting the lysis of LukED to cells, we simply explored the cytotoxicity of LukED and the protective effect of scFv on HEK293T cells containing different chemokine receptors. The results showed that the purified LukED, rather than LukE or LukD, lysed HEK293T cells containing CCR5 and CXCR2 in a dose and time-dependent manner (Figure 3A–D). The results are consistent with the previous studies [12,16,41]. Compared with the LukED group (10 μg/mL), the dead cell rate of the HEK293T cells containing CCR5 (from 80% to 20%) or CXCR2 (from 80% to 40%) was significantly reduced in the scFvs mixed group, exhibiting 4-fold and 2-fold decreases, respectively (*p* < 0.05). Meanwhile, HEK293T cells containing CCR5 and CXCR2, rather than CXCR1, can be lysed by LukED (Figure 3E), indicating that the purified LukED can recognize CCR5 and CXCR2 expressed on the cell membrane, thus resulting in cell death. The results are consistent with the previous studies [12]. Vrieling et al. reported that anti-LukM could significantly inhibit the lysis of bovine neutrophils by LukMF [12,15]. Similarly, the cytolysis of LukED can be inhibited by scFvs (Figure 3A–E), suggesting that the scFvs obtained in this experiment might inhibit the binding of LukED to CCR5 and CXCR2 through competitive binding of LukED; thus, protecting the integrity of the cell membrane. Next, we will further verify whether the interaction between the CDR3 of scFv and LukED inhibits the binding of LukED to the receptors on the surface of the cell membrane, thus weakening the lytic effect of LukED on cells.

Then, the cytotoxicity assay on bovine neutrophils and MAC-T cells was performed to evaluate the lysis ability of LukED, as well as the protective effect of scFv. The results revealed that the purified LukED had an obvious cytotoxic effect on bovine neutrophils and MAC-T cells, and the cell death rate increased with the rise of LukED concentration. The results were similar to the previous studies [12,15], suggesting that the receptors on the surface of bovine neutrophils and MAC-T cells might be recognized by LukED; thus, resulting in cell death. It was noticed that the scFvs mixture group could significantly reduce the death rate of bovine neutrophils and MAC-T cells compared with the LukED group (greater than 0.312 μg/mL) (*p* < 0.05). Meanwhile, the results showed that scFvs protected bovine neutrophils and MAC-T cells from killing by LukED in a dose-dependent manner (Figure 4C). These results also suggested that scFv might competitively bind to LukED, thus inhibiting the binding of LukED to the receptors on the surface of bovine neutrophils and MAC-T cells, thereby protecting cells from lysis. In addition, the cell-killing effect on bovine neutrophils and MAC-T cells in the scFvs mixture group was significantly lower than that of LukED group (decreased from 80% to 17% in the bovine neutrophils group and from 43% to 12% in the MAC-T cells group) (*p* < 0.0001), as well as the ZL8 + ZL42 and ZL22 + ZL23 groups (*p* < 0.05). We speculate that the molarity of the scFvs mixture group was not enough to bind to LukE and LukD completely so that the protective effect of the scFvs mixture group was better than that of the ZL8 + ZL42 and ZL22 + ZL23 groups.

Because the true biological activity of LukED secreted by *S. aureus* may be different from the activity of recombinant LukED produced in *E. coli*, three *S. aureus* strains (USA300, ATCC25923 and XD69) and their supernatants were used to detect the lytic effect on bovine neutrophils and MAC-T cells. The results indicated that the cytotoxicity of the scFvs mixture group to bovine neutrophils and MAC-T cells at different time points was significantly lower than that of the USA300, ATCC25923 or XD69 groups (*p* < 0.05) (Figure 5A–D). Furthermore, the cell death rates of bovine neutrophils (decreased from 90% to 60% in the USA300 group, from 80% to 45% in the ATCC25923 group and from 90% to 55% in the XD69 group) and MAC-T cells (decreased from 25% to 15% in the USA300 group, from 16% to 8% in the ATCC25923 group and from 26% to 16% in the XD69 group) reduced significantly after a 6-h incubation with the scFvs and the supernatants of *S. aureus*. Some studies have shown that *S. aureus* could lyse more than 80% of the MAC-T cells, and the supernatant of *S. aureus* could also lyse more than 80% of the bovine neutrophils, the results of which were similar to the present study [12,42]. The cell-killing effect of the USA300 and XD69 groups on MAC-T cells or bovine neutrophils was stronger than that of the ATCC25923 group, indicating that the expression level of LukED might be different; thus, resulting in different lethality among various strains. The overall results indicated that scFvs could inhibit the cytotoxicity of *S. aureus* to MAC-T cells and bovine neutrophils, thereby providing a potential protective effect on bovine mastitis caused by *S. aureus*.

The bacterial proliferation assay was performed to evaluate the specific contribution of *S. aureus* on mammary gland tissue. The results showed that three *S. aureus* strains could facilitate colonization and bacterial proliferation in mammary gland tissue infected systemically by three *S. aureus* strains (Figure 6A–D). Compared with the USA300, ATCC25923 and XD69 groups, the scFvs mixture group could significantly reduce the bacterial burden seeded in mammary gland tissue from 12 h after infection (Figure 6A). After a 72-h infection, the bacterial burden was 1000 times higher than that at 12 h (from 1 × 10^5^ to 1 × 10^8^ cfu/mL) in the NC groups (Figure 6D). Studies have shown that LukED contributes to the virulence of *S. aureus* by facilitating the proliferation of *S. aureus* within seeded tissue, which is consistent with this research [5]. Meanwhile, the bacterial burden in the scFvs mixture group only increased by 100 times (from 3.2 × 10^5^ to 3.2 × 10^8^ cfu/mL) from 12 to 72 h, and the inhibition effect of the scFvs mixture group was better than that of the ZL8 + ZL42 and the ZL22 + ZL23 groups (*p* < 0.05) (Figure 6B,D), indicating that scFv probably weakened the proliferation of *S. aureus* in vivo by inhibiting the effect of LukE and LukD, especially the scFvs mixture.

The pathological damage of mammary gland tissue was observed by H&E staining and via microscope. The results showed that there were no histopathological changes in the control group (Figure 7A). Severe pathological changes were observed in the NC group, such as the destruction of mammary epithelial cells and mammary acini, the rupture of the mammary lobule and the infiltration of inflammatory cells (Figure 7B), suggesting that the inflammation has occurred in the mammary gland tissue infected by *S. aureus*. These results are similar to the previous studies [8,43,44,45]. The histological changes were improved obviously in the scFvs mixture group, exhibiting a similar protective effect to the PC group and showing mild inflammatory injury and slight infiltration of inflammatory cells (Figure 7C,F). In addition, the MPO activity assay also showed similar results (*p* < 0.05) (Figure 7H), suggesting that scFvs could decrease the inflammatory response caused by *S. aureus*; thus, weakening the chemotaxis of bovine neutrophils.

The production level of pro-inflammatory cytokines can reflect the severity of inflammation caused by *S. aureus*. Previous studies have shown that TNF-α can induce the cascade release of other inflammatory cytokines, such as IL-6 [46,47]. IL-6 and IL-1β can regulate the host immune response induced by *S. aureus* and recruit inflammatory cells (such as bovine neutrophils) to the lesion area; thus, causing a series of inflammatory responses [48,49,50]. The results showed that the transcription and expression levels of the five pro-inflammatory cytokines in the NC groups were significantly higher than those in the control group, verifying the previous studies. Studies also reported that IL-8 and IL-18 can promote neutrophil chemotaxis and play an important role in anti-bacterial immune response [51,52,53]. The expression levels of TNF- α (from 490 to 190 pg/mL), IL-18 (from 890 to 300 pg/mL), IL-6 (from 540 to 200 pg/mL), IL-1 β (from 780 to 270 pg/mL) and IL-8 (from 480 to 150 pg/mL) in mammary gland tissue in the scFvs mixture group were significantly decreased compared to the NC group, indicating that scFvs have an anti-inflammatory effect on the process of inflammatory responses. Additionally, the MPO activity (decreased from about 1.22 to 0.78 U/g) assay also verified the anti-inflammatory effect of scFvs, exhibiting similar results to previous studies [8,54]. We speculate that scFvs weakened the virulence of *S. aureus* and reduced the damage of mammary gland tissue, thus decreasing the transcription and expression levels of five pro-inflammatory cytokines. These studies demonstrated that appropriate levels of pro-inflammatory cytokines are essential for host defenses, while excessive secretion can lead to systemic inflammation and damage rather than protection.

## 4. Materials and Methods

### 4.1. S. aureus Strains, Plasmid and Cell Culture

Three *S. aureus* strains, namely USA300, ATCC25923 and XD69, were used in this study. These strains were cultured aerobically in Tryptic Soy Broth (TSB) at 37 °C until the OD_600_ reached about 0.6. MAC-T cells and HEK293T cells were cultured in RPMI 1640 and DMEM basic (1×), respectively, supplemented with 10% fetal bovine serum (FBS) maintained at 37 °C with 5% CO_2_, no penicillin and streptomycin were added during incubation with *S. aureus* unless stated otherwise [8]. USA300 strain and MAC-T cells were kindly provided by professor Jianhe Sun, Shanghai Jiao Tong University, China. ATCC25923 strain and HEK293T cells were preserved in our lab. XD69 was isolated from Shanghai Xi Di dairy farm, China. The plasmids of pET-28a (+), pGEX-4T-1 and pEGFP-N1, were preserved in our lab.

### 4.2. Protein Preparation of LukED

Firstly, the genes of *lukE* and *lukD* were amplified by using the template of the genomic DNA of *S. aureus* strain ATCC25923. The primers used to amplify the two genes are shown in Table 3. Secondly, the PCR products of *lukE* and *lukD* genes were linked to the pET-28a (+) plasmid via double enzyme digestion (*Bam*HI and *Xho*I). Thirdly, the two recombinant plasmids, namely pET-28a-LukE and pET-28a-LukD, were transformed into DH5α and BL21 (DE3) competent *Escherichia coli* (*E. coli*) cells (Invitrogen, Carlsbad, CA, USA) successively [55]. After sequencing, the competent cell was cultured in Luria-Bertani (LB) at 37 °C until the cultures reached an OD_600_ of 0.6. Then, the cultures were induced with 0.6 mM IPTG (Sigma-Aldrich, St. Louis, MO, USA) at 16 °C for 16 h. The bacterial cell pellets were resuspended with phosphate-buffered saline (PBS) buffer and then were lysed by sonication on ice for 40 min. [56]. Afterward, the soluble proteins were purified with a Ni-NTA His-Bind Resin column (Merk, Madison, WI, USA) according to the manufacturer’s instructions. The proteins were eluted with imidazole in different concentrations successively and then were detected by 10% SDS-PAGE. The protein concentrations was measured by the BCA Protein Assay Kit (Sangon Biotech, Shanghai, China) [25].

### 4.3. Affinity Selection of ScFvs against LukE and LukD

The scFv phage display library has been constructed and optimized in our lab previously. In this study, the purified proteins of LukE and LukD were used as the coating antigens. The optimized phage library targeting LukE and LukD was enriched after five rounds of bio-panning. The bacterial clones were randomly selected from the phage library after the final round of bio-panning. Each clone was infected with the M13KO7 helper phage for the acquisition of recombinant phages, and the preparation of the recombinant phage was described in the previous section. Afterward, phage ELISA was performed to select the positive clones that had the binding affinity with the two protein antigens. Similarly, the proteins of LukE and LukD were used as the antigens, and the recombinant phages were incubated on the 96-well plate. M13KO7 helper phage was used as the negative control. The procedures of phage ELISA for the selection of positive clones can be referenced from previous studies [8,29].

### 4.4. Protein Purification of ScFvs Targeting LukE and LukD

Four positive clones obtained by phage ELISA were used as the template to amplify the scFvs targeting LukE and LukD. The primers for amplification of the four scFvs were identical as below: forward primer containing *Eco*RI (underlined): 5′-CCGGAATTCATGGCCCAGGCTGTGCTGACTCAG and reverse primer containing *Xho*I (underlined): 5′-CCGCTCGAGACTAGTGGAGGAGACGGTGAC. The four scFvs, namely ZL8, ZL42 (targeting LukE) and ZL22, ZL23 (targeting LukD), were linked to the pGEX-4T-1 vector carrying the N-GST tag. Afterward, the recombinant plasmids were transformed into DH5α and BL21 (DE3)-competent *E. coli* cells for protein expression [40]. The purification of four soluble scFv proteins with the N-GST tag was performed with the GST-Sefinose ™ Kit (Sangon Biotech, China) according to the manufacturer’s instructions. The scFv proteins were stored at −80 °C until use.

### 4.5. Plasmid Construction of Bovine Chemokine Receptor

The peripheral blood lymphocytes (PBLs) of a cow with *S. aureus*-induced mastitis was acquired from the Shanghai Xi Di dairy farm. Total RNA extracted from PBLs with TRIzol Reagent (Invitrogen, USA) was transcribed to cDNA using a reverse transcription kit (Takara Qingdao, China). The primers used to amplify the chemokine receptor genes (CCR5, CXCR1 and CXCR2) are shown in Table 4. The genes of the chemokine receptor were amplified from bovine cDNA by PCR and then subcloned into the pEGFP-N1 plasmid for stable expression [12].

### 4.6. Cell Transfection

HEK293T cells were cultured in DMEM basic (1×) supplemented with 10% FBS, 100 U/mL penicillin and 100 μg/mL streptomycin and maintained at 37 °C with 5% CO_2_. Lipofectamine 3000 (Life Technologies) was used to transfect the plasmids containing chemokine receptors into the HEK293T cells according to the manufacturer’s instructions; the method of cell transfection can be referenced from previous studies. The empty pEGFP-N1 plasmid and CXCR1 were used as the control [15].

### 4.7. Cell Viability Assay

A cell viability assay was performed to evaluate the viability of HEK293T cells containing CCR5 and CXCR2 after infection with LukED. The specific steps are carried out in accordance with the manufacturer’s instructions of the CellTiter-Glo^®^ Luminescent Cell Viability Assay (Promega, Madison, WI, USA). After cell transfection, the cells were intoxicated with LukE, LukD or a mixture of LukE+LukD in different concentrations at a range of time points, and the mixture of the four scFvs was added simultaneously to the plate in a final concentration of 40 μg/mL. The control (C) group contained 100 μL of RPMI 1640 supplemented with 10% FBS. A total of 0.1% *v*/*v* Triton X-100 was used as a positive control (PC) for 100% cell death. After the intoxication, CellTiter Glo reagent was added to each well for an additional 1 h incubation at 37 °C, 5% CO_2_. Then, the value of Ab_492_ for colorimetric reaction were measured by using the Perkin Elmer Envision 2103 Multilabel Reader. The calculation methods of percent viable cells can be referenced from previous studies [57,58].

### 4.8. Leukocyte Isolation

The bovine blood was acquired from a healthy dairy cow in Shanghai Xi Di dairy farm by using a sterile blood collection system with EDTA anti-coagulant (BD Vacutainer). Percoll (1.09176 g/L) centrifugation was performed to isolate the neutrophils from bovine blood according to the previous studies. The viability and purity of the bovine neutrophils were ≥95% [15,59].

### 4.9. LDH Cytotoxicity Assay

To evaluate the LukED-mediated killing of bovine neutrophils and MAC-T cells in vitro and the protective effect of scFvs, an LDH cytotoxicity assay was performed by using the LDH Assay Kit (Abcam, Cambridge, UK) according to the manufacturer’s instructions. In brief, 1 × 10^5^ per well of MAC-T cells were cultured in a 96-well plate at 37 °C, 5% CO_2_. Then, cells were intoxicated with 1 × 10^6^ cfu/mL of *S. aureus* (MOI = 10) or the recombinant proteins (LukE, LukD or a mixture of LukE + LukD in different concentrations) diluted with RPMI 1640 supplemented with 1% FBS at a range of time points, and the mixture of four scFvs was added simultaneously to the plate in a final concentration of 40 μg/mL. The negative control group (NC group) contained 200 μL of RPMI 1640 supplemented with 1% FBS. One hour before the scheduled detection time point, LDH release reagent (10% volume of original cell culture medium) was added to the “sample maximum enzyme activity control well” as the positive control group (PC group). After the infection, the 96-well plate was centrifuged at 400× *g* for 5–10 min. Subsequently, a 120 μL aliquot of the cell culture medium was transferred to a new plate, then 60 μL of detection kit reaction mixture was added to the plate. After a 30-min incubation at the ambient temperature without light, the value of Ab_490_ was measured with a microplate reader. The cytotoxicity rate was calculated in accordance with the specification [60].

### 4.10. Animal Experiment

For in vivo experiments, after parturition for 10 days, adult female BALB/c mice were randomly divided into four groups (control group, NC group, PC group and scFvs mixture group). Each group was intoxicated with three *S. aureus* strains (USA300, ATCC25923 and XD69). The mouse mastitis model was established as described previously [25]. All kinds of the solution were injected subcutaneously into the teats of the R4 and L4 of the mammary gland tissues. Afterward, the control group and NC group were treated with 100 μL of physiological saline. The PC group was treated with 100 μL of 100 mg/mL penicillin, while the scFvs mixture group was treated with the mixture of four scFvs (20 mg/Kg). Six hours later, 100 μL of physiological saline was injected into the teats of the control group, while 100 μL of 10^8^ cfu/mL *S. aureus* (USA300, ATCC25923 and XD69) were injected into the teats of the NC, PC and scFvs mixture groups. Finally, all mice were euthanized with CO_2_ inhalation at 48 h post-inoculation after anesthetization with sodium pentobarbital (40 mg/kg). The mammary gland tissues and blood samples were collected for assessment of the following experimental indicators [26].

### 4.11. Bacterial Proliferation of Mammary Gland Tissues

A bacterial proliferation assay was performed to evaluate the specific contribution of LukED and the protective effect of scFvs. Firstly, 100 μL of 10^8^ cfu/mL *S. aureus* and the four scFvs targeting LukE and LukD were pre-mixed for 30 min. Then, the mixture was injected subcutaneously into the teats of the R4 and L4 of the mammary gland tissues. After the infection for 12, 24, 48 and 72 h, a 100 mg aliquot of mammary gland tissues were collected and homogenized in PBS and then incubated onto tryptic soy agar (TSA) plates at 37 °C through serial dilutions. A bacterial proliferation assay was performed with each group of six mice [5].

### 4.12. Histopathological Evaluation of Mammary Gland Tissues

*S. aureus*-induced mastitis in mice can cause severe infiltration of neutrophils and macrophages in most areas and areas of mammary gland tissues. Histopathological evaluation of mammary gland tissues can reflect the pathogenicity of *S. aureus* and the protective effect of the scFv. After the infection, the mammary gland tissues were excised and fixed with 4% paraformaldehyde, dehydrated and embedded with paraffin wax. Then, the tissues were deparaffinized with xylene, and 4 μm thick slices were stained with hematoxylin and eosin (H&E). The samples were observed under an optical microscope (Olympus, Tokyo, Japan) [61,62]. The mammary gland tissues alternation index was performed to evaluate the histopathological changes as described in previous studies [43,44]. The damage level of mammary acini and mammary epithelial cells, the integrity of lobuli mammae, the inflammatory cells infiltration and the thickening of alveolus walls were considered as the indicators of histopathological changes. Each histological feature was graded 0 to 5 [44,61].

### 4.13. MPO Activity Assay

Polymorphonuclear neutrophils (PMNs) are the primary components of inflammation and immune response in bovine mastitis. A myeloperoxidase (MPO, existed in PMNs) activity assay was performed to evaluate the inflammatory response severity of the parenchymal infiltration of neutrophils and macrophages in mammary gland tissues. In brief, after the infection, a 100 mg aliquot of mammary gland tissues was homogenized and heated with reaction buffer (volume ratio 1:9). The detection steps were in accordance with the manufacturer’s instructions (Nanjing Jiancheng Bioengineering Institute, Nanjing, China). The value of OD_460_ was used to evaluate the MPO activity [26,45].

### 4.14. ELISA Assay

The expression level of five inflammatory cytokines (TNF-α, IL-18, IL-6, IL-1β, IL-8) was used to evaluate the inflammatory response of mammary gland tissues. The different groups of mammary gland tissues were collected and homogenized with PBS on ice. After centrifugation, the supernatants were harvested by centrifugation for the following assay. ELISA assay was performed to assess the expression level of the five inflammatory cytokines by ELISA kits (Multisciences, Hangzhou, China) according to the manufacturer’s instructions [43,63].

### 4.15. qPCR Assay

qPCR assay was performed to evaluate the transcription level of TNF-α, IL-18, IL-6, IL-1β and IL-8 in mammary gland tissues from *S. aureus*-induced mice mastitis. Total RNA was then extracted from the mammary gland tissues using the TRIzol reagent, and the cDNA was generated by reverse transcription using a reverse transcription kit (Invitrogen, China) according to the manufacturer’s instructions. The specific primers of five cytokines for the qPCR assay are listed in Table 5 [26,45,64,65]. An ABI 7300 Real-Time PCR Detection System (Applied Biosystems, Foster, CA, USA) was used for qPCR assay. Each sample was assessed in triplicate in a 20-μL reaction volume. The reaction component and the reaction condition can be referenced from the studies previously in our lab [29]. The results (fold changes) were normalized by GAPDH using the 2^−ΔΔCt^ comparative method [45].

### 4.16. Statistical Analysis

All tests of the statistical analyses were performed on GraphPad Prism 6 software (San Diego, CA, USA) and SPSS 18.0 software (SPSS, Inc., Chicago, IL, USA). The data were expressed as mean ± standard error, and the intergroup differences were assessed by multiple comparisons of variance (one-way analysis of variance). The differences were considered statistically significant when *p* < 0.05.

## 5. Conclusions

In summary, four scFvs targeting LukE (ZL8 + ZL42) and LukD (ZL22 + ZL23) were selected using phage display techniques in this study. These scFvs attenuated the lytic effect of LukED on cells by competitively binding to LukED with cell surface receptors (CCR5 and CXCR2). Meanwhile, studies have shown that scFvs against LukE and LukD could inhibit the killing effect of *S. aureus* strains and their supernatant on MAC-T cells and bovine neutrophils. In addition, histopathological observation and detection of pro-inflammatory cytokines showed that scFvs could alleviate the inflammation of *S. aureus*-induced mastitis in mice, suggesting that scFvs may have a potential protective effect on bovine mastitis caused by *S. aureus*. This study provides a basis for further study on the pathogenic mechanism of virulence factors and the prevention and treatment of bovine mastitis caused by *S. aureus*. The molecular mechanism of the anti-inflammatory effect of scFv will be deeply studied in the future.

## Figures and Tables

**Table 1:** The enrichment of scFv phage display library targeting LukE.

Round of Screening	Input (cfu/mL)	Output (cfu/mL)	Output /Input	Enrichment Fold	Total Enrichment Fold
1	1.00 × 10^13^	1.65 × 10^6^	1.65 × 10^−7^	1	
2	1.00 × 10^13^	2.77 × 10^6^	2.77 × 10^−7^	1.68	
3	1.00 × 10^13^	8.67 × 10^6^	8.67 × 10^−7^	3.13	
4	1.00 × 10^13^	6.25 × 10^7^	6.25 × 10^−6^	7.21	
5	1.00 × 10^13^	2.14 × 10^8^	2.14 × 10^−5^	3.42	129.70

**Table 2:** The enrichment of scFv phage display library targeting LukD.

Round of Screening	Input (cfu/mL)	Output (cfu/mL)	Output /Input	Enrichment Fold	Total Enrichment Fold
1	1.00 × 10^13^	4.25 × 10^5^	4.25 × 10^−8^	1	
2	1.00 × 10^13^	1.32 × 10^6^	1.32 × 10^−7^	3.11	
3	1.00 × 10^13^	4.24 × 10^6^	4.24 × 10^−7^	3.21	
4	1.00 × 10^13^	6.06 × 10^7^	6.06 × 10^−6^	14.29	
5	1.00 × 10^13^	1.25 × 10^8^	1.25 × 10^−5^	2.06	294.12

**Table 3:** The primers of *lukE* and *lukD* genes for amplification.

Primer Name	Sequence (5′-3′)	Size (bp)	Reference
LukE-F	CCCGGATCCAATACTAATATTGAAAATATTGGTGATG	849	This study
LukE-R	CCGCTCGAGTTAATTATGTCCTTTCACTTTAATTTCG		
LukD-F	CGCGGATCCGCTCAACATATCACACCTGTAAGC	906	This study
LukD-R	CCGCTCGAGTTATACTCCAGGATTAGTTTC		

**Table 4:** The primers of chemokine receptor genes for amplification.

Primer	Sequence (5′-3′)
CCR5-F	CCGCTCGAGGCCACCATGGATTATCAAACATCAACTCCCCTCTA
CCR5-R	CCCAAGCTTCAAGCCAACAGAGATTTCCTGTTCTC
CXCR1-F	CCGCTCGAGGCCACCATGGTTGGTGACTCAGTCTTTCAACC
CXCR1-R	CCCAAGCTTGAGGGTAGTAGACGTGTTCCCTGAA
CXCR2-F	CCGCTCGAGGCCACCATGGCTGAAACAAAATTTACTTCAAATATAGAAGGA
CXCR2-R	CCCAAGCTTGAGGGTAGTAGACGTGTTCCCTGA

**Table 5:** The primers of five cytokines for qPCR assay.

Primer Name	Sequence (5′-3′)	Size (bp)	Reference
IL-18-F	TGGTTCCATGCTTTCTGGACTCCT	132	[8]
IL-18-R	TTCCTGGGCCAAGAGGAAGTGATT		
TNF-α-F	GCCTCCCTCTCATCAGTCTA	223	[8]
TNF-α-R	GGCAGCCTTGTCCCTG		
IL-6-F	AGTTGTGCATGGCAATTCTGA	213	[8]
IL-6-R	AGGACTCTGGCTTGTCTTTCT		
IL-1β-F	ACCTGTGTCTTCCCGTGG	171	[8]
IL-1β-R	TCATCTCGAGCCTGTAGTG		
IL-8-F	CGGCAATGAAGCTTCTGTAT	224	[8]
IL-8-R	CCTTGAAACTCTTTGCCTCA		
GAPDH-F	CAATGTGTCCGTCGTGGATCT	124	[8]
GAPDH-R	GTCCTCAGTGTAGCCCAAGATG		

## Data Availability

The datasets generated and analyzed during the current study are available from the corresponding author on reasonable request.
